# Clinical impact of complement (C1q, C3d) binding *De Novo* donor-specific HLA antibody in kidney transplant recipients

**DOI:** 10.1371/journal.pone.0207434

**Published:** 2018-11-14

**Authors:** Hyeyoung Lee, Eunhee Han, Ae-Ran Choi, Tae Hyun Ban, Byung Ha Chung, Chul Woo Yang, Yeong Jin Choi, Eun-Jee Oh

**Affiliations:** 1 Department of Laboratory Medicine, Seoul St. Mary’s Hospital, College of Medicine, The Catholic University of Korea, Seoul, Korea; 2 Department of Laboratory Medicine, International St. Mary's Hospital, College of Medicine, Catholic Kwandong University, Incheon, Korea; 3 Division of Nephrology, Department of Internal Medicine, Seoul St. Mary’s Hospital, College of Medicine, The Catholic University of Korea, Seoul, Korea; 4 Department of Hospital Pathology, Seoul St. Mary's Hospital, College of Medicine, The Catholic University of Korea, Seoul, Korea; Medical University of Gdansk, POLAND

## Abstract

**Introduction:**

Complement binding activity of donor-specific HLA antibodies (DSA) has been suggested as a new tool to stratify immunologic risk in kidney transplantation (KT). The objective of this study was to evaluate the clinical implication of C1q/C3d binding activity of *de novo* DSA (dnDSA) in KT recipients.

**Material and methods:**

A total of 161 pretransplant DSA-negative recipients were monitored for dnDSA at the time of biopsy. C1q/C3d binding activities of dnDSA were assessed using C1qScreen assay (One lambda, USA) and Lifecodes C3d detection assay (Immucor, USA), respectively. Clinical outcomes including biopsy-proven antibody mediated rejection (AMR), C4d detection and post-biopsy graft survival were investigated.

**Results:**

De-novo DSAs were detected in fifty-four (33.5%) patients (HLA class I only, n = 19; class II only, n = 29; both class I and II, n = 6). Of them, complement binding activities were detected in 26 (48.1%) patients, including 17 C1q+ and 24 C3d+ patients. Both C1q and C3d positivity were associated with increased mean fluorescence intensity values of dnDSA. Complement binding activity of dnDSA enhanced the incidence of AMR (25.0% in C1q-C3d-, 36.4% in C1q+/C3d- or C1q-/C3d+, and 60.0% in C1q+/C3d+ patients) (*P* <0.001). The incidence of AMR was not different between patients with C1q+ and those with C3d+ dnDSA (64.7%, 11/17 versus 45.8%, 11/24, *P* = 0.238). In comparison between C1q and C3d assay according to HLA specificity, C1q+ HLA class I ± II dnDSA was the best predictor for AMR (odds ratio: 27.2). C1q+/C3d+ dnDSA was associated with more C4d deposition in allograft tissue and inferior post-biopsy graft survival. Clinical outcomes were not significantly different between C1q+ and C3d+ dnDSA-positive patients.

**Conclusion:**

Detection of complement binding activity using both C1q and C3d assays can be a further prognostic marker for predicting AMR and allograft outcome in dnDSA+ kidney transplant patients.

## Introduction

Even with the development of strong immunosuppressive regimens, antibody mediated rejection (AMR) is a leading cause of long term kidney allograft loss [1]. Detection of antibodies against donor specific HLA antigens (DSA) initiates sequence of immune injuries in AMR [2]. Single antigen bead (SAB) assay based on Luminex platform has become a standard tool for detecting DSA in transplant patients [3, 4]. *De novo* DSA (dnDSA) can detect about 7–29% of kidney transplant recipients when it is tested with SAB [5, 6]. It has been reported that dnDSAs are associated with acute and chronic antibody mediated rejections [7–10], chronic graft dysfunction [9], and low allograft survival [11, 12].

Although DSAs can induce a wide range of graft injuries from no damage to severe rejection, as not all DSAs are responsible for causing AMR or for the inferior outcome of transplant [2, 13, 14]. Since improved analysis is needed to better distinguish clinically relevant DSA, SAB assays for detecting complement-binding activity of HLA antibodies (C1q, C3d) have been introduced [15, 16] with the hypothesis that complement binding antibodies are more harmful to the graft compared with their non-activating counterparts [2]. C1q is the first component in the classic complement pathway and C3d is a cleavage product of C3 in the downstream of complement cascade. Several studies have shown the association of C1q or C3d binding DSAs with AMR or poor graft survival [15–22]. Although several studies have compared two complement-binding assay [15, 22–24], their results are controversial as the superiority or difference of diagnostic utility remains unclear. The objective of this study was to test both C1q and C3d binding activities of dnDSA at the time of biopsy and investigate histopathological and clinical impact of them in kidney transplant patients.

## Materials and methods

### Study population

**Fig 1:**
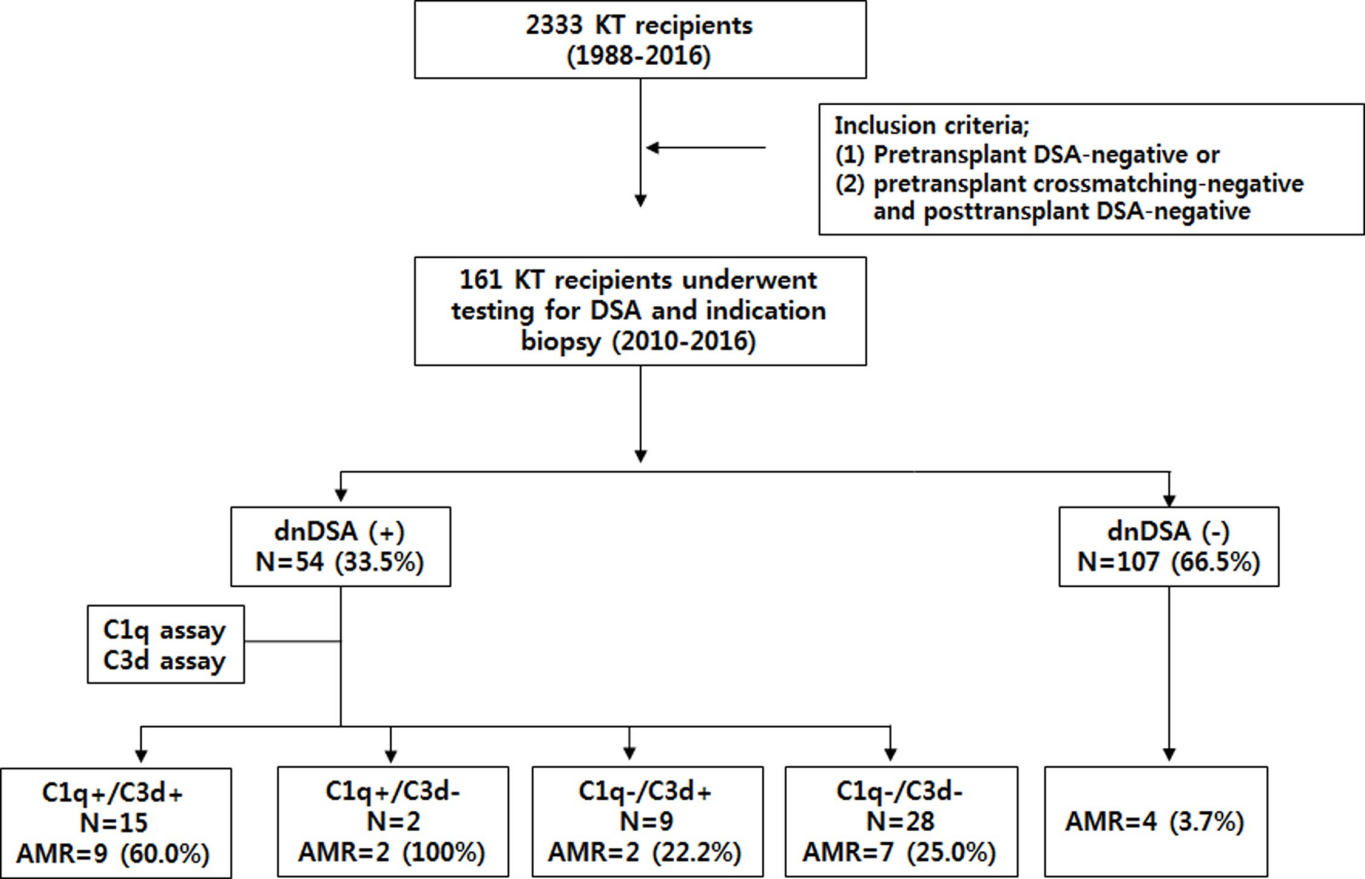
Study design. Abbreviation: dnDSA, de-novo donor specific HLA antibody; AMR, antibody mediated rejection.

Among 2,333 patients who received renal transplants between March 1988 and February 2016, we included 161 adult recipients who were pretransplant DSA negative and underwent indication biopsy and DSA testing between February 2010 and May 2016 at Seoul St. Mary's hospital (Fig 1). The baseline characteristics are summarized in Table 1. The immunosuppressive regimen at our hospital has been previously described [25, 26]. Pretransplant desensitization was performed in four patients who received ABO-incompatible transplants and five patients with pre-transplant high PRA (≥ 50%) despite no DSA [27, 28]. Tacrolimus or cyclosporine was administered with mycophenolate mofetil and prednisolone for initial immunosuppressant. Basiliximab was administered as an additional induction therapy. Indication biopsy was performed for patients with an increase in serum creatinine level (20% above baseline value) or new-onset of proteinuria. The median time to biopsy was 8.0 months (interquartile range: 1.5–64.3) after KT. And the median follow-up time after biopsy was 47.7 (interquartile range: 24.7–63.9) months. This study was approved by the Institutional Review Board of Seoul St. Mary’s Hospital (KC13TNMI0701). Of 161 recipients, informed written consents were obtained from 128 patients. For 33 patients who received KT before January 2013, informed consent was waived by the board (KC10SISI0057) because the current retrospective study was performed using medical records and leftover serum specimen.

**Table 1:** Clinical characteristics of 161 patients.

Characteristics	N = 161
Age at transplantation (year, mean (SD))	42.2 (11.9)
Gender; male, n (%)	124 (77.0)
Re-transplantation, n (%)	7 (4.3)
Deceased donor, n (%)	55 (34.2)
Donor gender; male, n (%)	72 (44.7)
Donor age(year, mean (SD))	44.0 (12.7)
HLA mismatch, n (mean (SD))	3.2 (1.6)
ABO incompatible, n (%)	4 (2.5)
Blood type, n (%)	
A	46 (28.6)
B	48 (29.8)
O	40 (24.8)
AB	27 (16.8)
Desensitization, n (%)	9 (5.6)
Immune suppressant, n (%)	
Cyclosporin,	37 (23.0)
Tacrolimus	124 (77.0)
Time since dialysis (month, median)(n = 138)	25.9
Post-KT months to biopsy, (month, median)	8.0
Pre-KT PRA (n = 122)	
HLA class I	
10–50%	11 (6.8)
>50%	2 (1.2)
HLA class II	
10–50%	8 (5.0)
>50%	7 (4.3)
DSA at the time of biopsy	
HLA class I only	19 (11.8)
HLA class II only	29 (18.0)
HLA class I + II	6 (3.7)
C1q +	17 (10.6)
C3d +	24 (14.9)
C1q + C3d +	15 (9.3)
Biopsy-proven AMR, n (%)	24 (14.9)
Graft failure, n (%)	44 (27.3)
Primary renal disease, n (%)	
Chronic glomerulonephritis	62 (38.5)
DM	26 (16.1)
HTN	25 (15.5)
ADPKD	3 (1.9)
Others	9 (5.6)
Unknown	36 (22.4)

Abbreviations: KT, Kidney transplantation; PRA, Panel reactive antibody; DM, Diabetes mellitus; HTN, Hypertension; ADPKD, Autosomal dominant polycystic kidney disease; AMR, antibody mediated rejection; dnDSA, de-novo donor specific HLA antibody

### Detection of donor specific HLA antibody and complement-binding activity

DSA was detected by using LABScreen Single Antigen (One Lambda Inc., A Thermo Fisher Scientific Brand, Canoga Park, CA, USA) as described in our previous report [6]. A mean fluorescence intensity (MFI) = 1,000 was used as a cut-off for positive results. Since 2009 we have been testing kidney recipients pretransplant and posttransplant for DSA using SAB assay. Therefore, for 42 patients who received transplants before 2009, we included the patients with a negative pretransplant T- and B-cell flow cytometric crossmatch and had negative DSA result in posttransplant monitoring. All 119 patients (73.9%) who received transplants since January 2009 were continuously followed DSA monitoring (1–3, 6, 12 months post-transplant and annually thereafter). DSA was assigned for HLA-A, HLA-B, HLA-C, HLA-DR, and HLA-DQ antigens. For all dnDSA (+) sera, both C1q and C3d assays were performed using C1q Screen^TM^ (One Lambda, CA, USA) and Lifecodes C3d detection (Lifecodes C3d detection, Immucor Transplant Diagnostics, Inc., Stamford, CT, USA), respectively. All tests were performed according to the manufacturer’s protocol. Beads with MFI of greater than 1000 were defined as positive in C1q and C3d assays, after considering the MFI values in C1q or C3d positive cases and previous report [24]. Prozone effect was suspected based on antibody response patterns and inconsistency between PRA screening test and SAB assay. In eight samples, SAB assays were retested after pretreatment with dilution, ethylenediaminetetraacetic acid (EDTA), dithiothreitol (DTT), or heat inactivation.

### Diagnosis and treatment of allograft rejection

These biopsies were performed using a 16-gauge biopsy gun under ultrasonic localization as described in our previous study [29]. Based on Banff working classification, histopathological diagnosis was made [30]. To detect C4d deposition, indirect immunofluorescence staining was performed with monoclonal anti-complement protein C4d antibodies (Biogenesis, Poole, UK; dilution1:50). C4d positivity was defined as C4d score ≥2 (focal or diffuse). Treatment of allograft rejection was as follows. When T cell mediated rejection was diagnosed, 3 to 5 daily boluses of intravenous methyl prednisolone (250 mg/day) were used followed by 5 to 7 days of oral steroid taper. Anti-thymocyte globulin was used for steroid therapy resistance. In case of AMR, plasmapheresis with intravenous immunoglobulin (IVIg) was used while rituximab was added in patients who did not respond. Response to AMR treatment was defined as negative for AMR at follow-up biopsy, reduction of serum creatinine level to ≤ 110% of baseline level and decreased proteinuria. In patients with chronic AMR, rituximab and IVIg for 4 days and IV methylprednisolone (250 mg per bolus) twice daily for 3 days followed by oral steroid taper for 5 to 7 days were applied.

### Statistical analysis

Results are described as mean ± SD or median and range for continuous variables. For categorical data, results are described as number and percentages. Comparisons of proportions among multiple subgroups were performed with Chi-square trend tests while comparisons between groups were performed using Mann–Whitney test. The concordance between C1q and C3d assay was evaluated by Cohen`s kappa coefficient. Graft loss was defined by the return to the long term dialysis, graft removal or patient’s death caused by graft related problem. Post-biopsy graft survival functions, censoring for death were compared using Kaplan-Meier analysis with log rank test. Multivariate analysis for graft survival were conducted using the Cox proportional hazards regression model. We included all variables with a P value <0.1 in the univariate analysis into multivariate analysis using a stepwise backward analysis, as previously proposed [31]. We further applied the competing risk regression as sensitivity analyses where death of patients from different causes was considered as a competing event (n = 161 patients, 44 graft failure and 12 deaths). Statistical analysis was performed using MedCalc version 15.5 (MedCalc, Mariakerke, Belgium), STATA version 15.1 (StataCorp LLC., College Station, TX) and R.2.4.0 statistical software. Results with P < 0.05 were considered statistically significant.

## Results

### Detection of dnDSA and complement binding activity

Of 161 recipients, dnDSA were detected in 54 (33.5%) patients at the time of biopsy. The majority of patients (53.7%, n = 29) had only HLA class II dnDSA while 35.2% (n = 19) had dnDSA against only HLA class I. However, 11.1% (n = 6) had both HLA class I and class II dnDSA. Overall, 25 patients had HLA class I dnDSA and 35 patients had class II dnDSA. In 54 patients with dnDSAs, 31.5% (n = 17) had C1q+ dnDSAs, 44.4% (n = 24) had C3d+ dnDSAs, and 51.9% (n = 28) had non-complement binding dnDSAs. Of 26 patients with C1q or C3d binding activities, majority (57.7%, 15/26) were both C1q and C3d positive (C1q+/C3d+), two (7.7%) patients had only C1q positive (C1q+/C3d-) dnDSAs, and nine (34.6%) patients had only C3d positive (C1q-/C3d+) dnDSAs. Regarding HLA specificity, patients with HLA class II dnDSAs had higher frequency of complement activity compared to patients with HLA class I dnDSA [60.0% (21/35) versus 24.0% (6/25), *P* = 0.006]. In comparison of two complement-binding assays, the overall concordance in 54 paired tests was 79.6% (k = 0.58). For HLA class I and HLA class II dnDSAs, concordance rates were 88.0% (k = 0.59) and 74.3% (k = 0.51), respectively. Regarding dnDSA beads detected, 54 patients had 71 dnDSAs, including 18 dnDSAs positive in C1q assays [HLA-A (n = 1), HLA-B (n = 4), HLA-C (n = 1), HLA-DQ (n = 11), HLA-DR (n = 1)] and 28 dnDSAs positive in C3d assays [HLA-A (n = 1), HLA-B (n = 3), HLA-DQ (n = 15), HLA-DR (n = 9)]. HLA-DQ was the most frequent dnDSA (61% of C1q+ dnDSA, 53.6% of C3d+ dnDSA) in both C1q and C3d assays. C3d assay showed higher positive rate in dnDSA against HLA-DR than C1q assay (32.1% vs 5.6%, *P* = 0.035).

### MFI values of dnDSA stratified by HLA class and complement-binding activity

**Fig 2:**
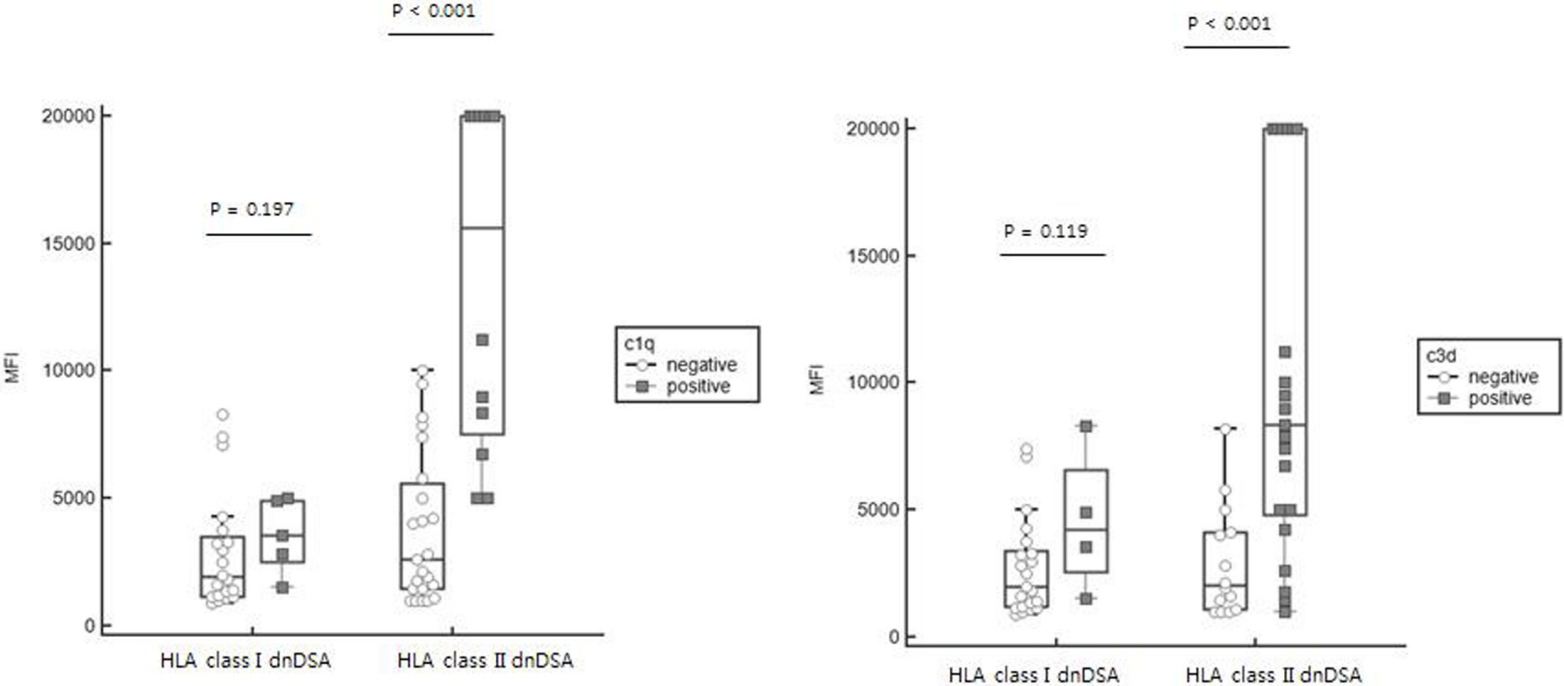
IgG MFI value according to HLA class and complement binding activity. MFI values of complement binding HLA class II DSAs were significantly higher than those of non-complement binding HLA class II DSAs in both C1q [median: 15,614 (interquartile range: 7,538–20,000) vs. median: 2,591 (1,446–5,613), *P* < 0.001] and C3d [median: 8,356 (4,804–20,000) vs. median: 2,035 (1,093–4,120), *P* < 0.001]. For the HLA class I DSAs, the difference of MFI values between complement binding and non-complement binding DSAs was not statistically significant in both C1q [median: 3,566 (interquartile range: 2,483–4,916) vs. median: 1,916 (1,170–3,522), *P* = 0.197] and C3d [median: 4,225 (2,533–6,584) vs. median: 1,997 (1,177–3,401), *P* = 0.119]. In the Box-and-whisker plot, the central box represents the values from 25 to 75 percentile and the middle line represents the median value. The horizontal line extends from a minimum value to a maximum value, excluding outside and far out values which are displayed as separate points. Abbreviation: dnDSA, de-novo donor specific HLA antibody; MFI, mean fluorescence intensity.

After correction for the prozone effect of suspected samples, MFI values of IgG-SAB assay between complement-binding and non-binding dnDSA were compared. Results according to HLA class specificity are shown in Fig 2. For HLA class II dnDSAs, both C1q and C3d binding dnDSAs had higher MFI values than C1q- or C3d- dnDSAs (*P* < 0.001 and *P* < 0.001, respectively). For HLA class II reactive dnDSAs, C1q+ dnDSAs had higher MFI values [median: 15,614 (interquartile range: 7,538–20000)] than C3d+ dnDSAs [median: 8,356 (4,804–20,000)] without statistical significance (*P* = 0.147). Regarding HLA class I dnDSAs, there were no significant differences in MFI values between C1q+ and C3d+ dnDSAs (*P* = 0.197 and *P* = 0.119, respectively). However, the median MFI of C1q assay (n = 17) was 19,973 (interquartile range: 8,478–26,302) and the median MFI of C3d assay (n = 24) was 19,882 (5,085–25,206).

### Clinical characteristics of dnDSA-positive patients

Clinical characteristics and clinical outcomes were compared according to results of C1q and C3d assays (Table 2). No significant differences were noted in recipient age, gender, type of transplantation, CKD etiologies (data not shown), donor gender, age, or between dnDSA+ and dnDSA- groups. Taking small numbers in the comparing groups into consideration, recipient age, donor age and immune suppressive regimen were different between C3d+ and C3d- patients. Patients with C3d + dnDSA showed higher level of proteinuria (at the time of biopsy) compared with C3d –dnDSA group at the time of biopsy (P = 0.039). C1q+ dnDSAs were associated with biopsy-proven AMR and graft failure with statistical significance (*P* = 0.005 and *P* = 0.026, respectively). C3d+ dnDSAs showed similar tendency of association without reaching statistical significance (*P* = 0.236 and *P* = 0.056, respectively).

**Table 2:** Comparison of clinical characteristics according to dnDSA, C1q and C3d positivity.

	dnDSA+	dnDSA-	C1q +	C1q -	C3d +	C3d -
	(n = 54)	(n = 107)	(n = 17)	(n = 37)	(n = 24)	(n = 30)
**Age at transplantation, year (mean, SD)**	42.6 (10.9)	42.0 (12.2)	39.9 (9.8)	43.9 (11.2)	38.9 ± 7.8	45.6 ± 12.1*
**Gender; male, n (%)**	41 (75.9)	83 (77.6)	15 (88.2)	26 (70.3)	18 (75.0)	23 (76.7)
**Re-transplantation, n (%)**	3 (5.6)	4 (3.7)	1 (5.9)	2 (5.4)	1 (4.2)	2 (6.7)
**Deceased donor, n (%)**	17 (31.5)	38 (35.5)	4 (23.5)	13 (35.1)	6 (25.0)	11 (36.7)
**Donor gender; male, n (%)**	20 (37.0)	52 (48.6)	8 (47.1)	12 (32.4)	10 (41.7)	10 (33.3)
**Donor age, year (mean, SD)**	42.9 (13.3)	44.7 (12.3)	40.8(13.9)	43.9 (13.1)	38.4 (12.4)	46.5 (13.0)*
**ABO incompatible, n (%)**	4 (7.4)	0 (0.0)†	1 (5.9)	3 (8.1)	1 (4.2)	3 (10.0)
**Desensitization, n (%)**	7 (13.0)	2 (1.9)†	2 (11.8)	5 (13.5)	1 (4.2)	6 (20.0)
**Immune suppressant, n (%)**						
Cyclosporin,	11 (20.4)	26 (23.6)	5 (29.4)	6 (16.2)	8 (33.3)	3 (10.0)*
Tacrolimus	43 (79.6)	81 (75.7)	12 (70.6)	31 (83.8)	16 (66.7)	27 (90.0)*
**Serum Cr at the time of biopsy, mg/dl (mean, SD)**	2.40 (1.35)	2.48 (1.71)	2.48 (1.35)	2.37 (1.37)	2.72 (1.71)	2.14 (0.93)
**Proteinuria at the time of biopsy, g/g (mean, SD)**	1.05 (1.16)	1.84 (3.61)	1.29 (1.26)	0.94 (1.11)	1.41 (1.44)	0.76 (0.78)*
**Biopsy-proven AMR, n (%)**	20 (37.0)	4 (3.7)†	11 (64.7)	9 (24.3)†	11 (45.8)	9 (30.0)
**Active AMR, n (%)**	6 (11.1)	2 (1.8)*	4 (23.5)	2 (5.4)	3 (12.5)	3 (10.0)
**Chronic active AMR, n (%)**	14 (25.9)	2 (1.9)†	7 (41.2)	7 (18.9)	8 (33.3)	6 (20.0)
**Graft failure, n (%)**	20 (37.0)	24 (22.4)*	10 (58.8)	10 (27.0)*	12 (50.0)	8 (26.7)

*, ^†^ Significantly different at:

*P < 0.05,

^†^ *P* < 0.005

Abbreviations: AMR, antibody mediated rejection; dnDSA, de-novo donor specific HLA antibody; Cr, creatinine

### Biopsy-proven AMR

**Fig 3:**
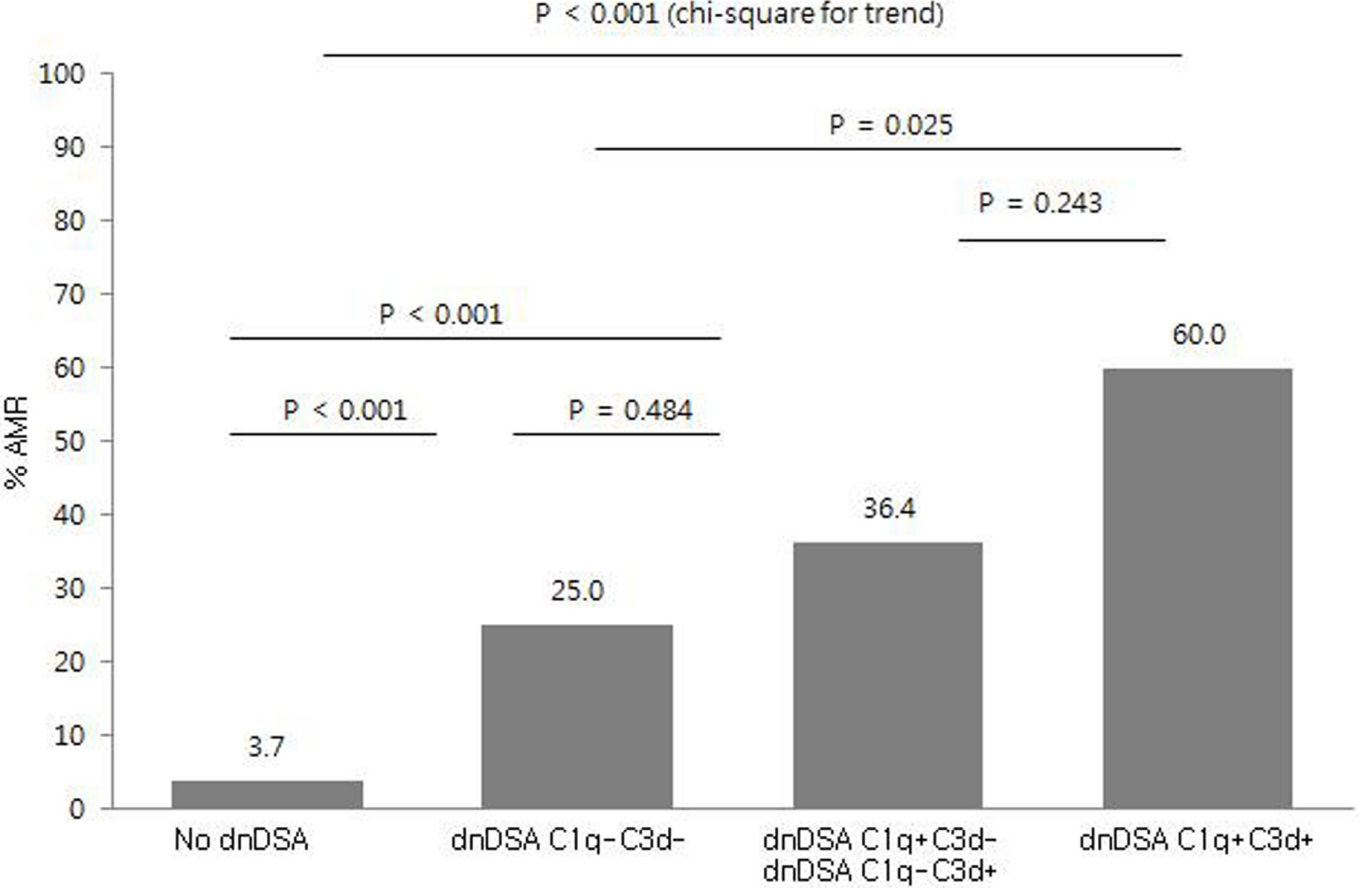
Ratio of antibody-mediated rejection according to complement binding activity. Complement binding activity of dnDSA enhanced the incidence of AMR (*P* < 0.001, Chi-square for trend). The comparison of each group was based on Fisher`s exact test. Patients with both C1q+ and C3d+ dnDSA had the highest frequency of AMR (9/15, 60.0%). Abbreviation: dnDSA, de-novo donor specific HLA antibody; AMR, antibody mediated rejection.

Of 161 patients who underwent allograft biopsy, twenty-four (14.9%) patients were diagnosed with biopsy-proven AMR. The incidence of AMR in patients with dnDSA was significantly higher than that in patients without dnDSA (37.0% versus 3.7%, *P* < 0.001). In 54 dnDSA positive patients, complement binding activity of dnDSA enhanced the incidence of AMR: 25.0% of patients with C1q-C3d- dnDSA, 36.4% of patients with C1q+/C3d- or C1q-/C3d+ dnDSA, and 60.0% of patients with C1q+/C3d+ dnDSAs (*P* < 0.001, Chi-square for trend) (Fig 3). The incidence of AMR was not significantly different between patients with C1q+ dnDSA and those with C3d+ dnDSA [64.7% (11/17) versus 45.8 (11/24), *P* = 0.238]. Six patients with C1q+ or C3d+ HLA class I dnDSA had higher incidence of AMR compared to 14 patients with C1q- C3d- HLA class I dnDSA [83.3% (5/6) versus 31.9% (6/19), *P* = 0.030]. In terms of HLA class II, AMR rates between patients with complement-binding class II dnDSA and those with non-complement-binding class II dnDSA were 42.9% (9/21) versus 14.3% (2/14) (*P* = 0.079).

As shown in Table 3, HLA class I ± II dnDSA, C1q+ HLA class I ± II dnDSA and C3d+ HLA class I ± II dnDSA had higher odds ratio than those for HLA-class II only. When we analyzed diagnostic performance of C1q or C3d assay in the study population, C1q-binding ability of dnDSA was a better predictor of AMR than dnDSA (odds ratio: 18.5 versus 15.2). The sensitivity and specificity of C1q+ dnDSA for AMR were 45.8% and 95.6%, respectively. Positive and negative predictive values of C1q+ dnDSA for AMR were 64.7% and 91.0%, respectively. C3d+ dnDSA also showed good specificity (90.5%) and NPV (90.5%). When we analyzed the diagnostic performance of C1q and C3d assays according to HLA class, complement-binding HLA class I ± II dnDSA showed higher odds ratio for the development of AMR (27.2 for C1q+ and 19.5 for C3d+ dnDSA) compared to HLA class II only dnDSA (11.1 for C1q+ and 5.3 for C3d+ dnDSA).

**Table 3:** Risk of AMR development in 161 kidney transplant patients.

	Odds ratio (95% CI)		P-value	Sensitivity	Specificity	PPV	NPV
**dnDSA**	15.2	(4.8–47.4)	<0.001	83.3	75.2	37.0	96.3
**C1q+**	18.5	(5.9–58.1)	<0.001	45.8	95.6	64.7	91.0
**C3d+**	8.1	(3.0–21.6)	<0.001	45.8	90.5	45.8	90.5
HLA class I ± II dnDSA	7.4	(2.8–19.7)	<0.001	45.8	89.8	44.0	90.4
**C1q+**	27.2	(2. 9–255.8)	0.004	16.7	99.3	80.0	87.2
**C3d+**	19.5	(1.9–195.6)	0.012	12.5	99.3	75.0	86.6
**HLA class II only dnDSA**	3.5	(1.4–9.1)	0.010	37.5	85.4	31.0	88.6
**C1q+**	11.1	(2.9–43.1)	<0.001	25.0	97.1	60.0	88.1
**C3d+**	5.3	(1.8–15.6)	0.003	29.2	92.7	41.2	88.2

Abbreviations: AMR, antibody mediated rejection; dnDSA: de novo donor-specific HLA antibodies; CI, confidence interval; PPV, positive predictive value; NPV, negative predictive value.

### C4d detection and histopathological findings

**Fig 4:**
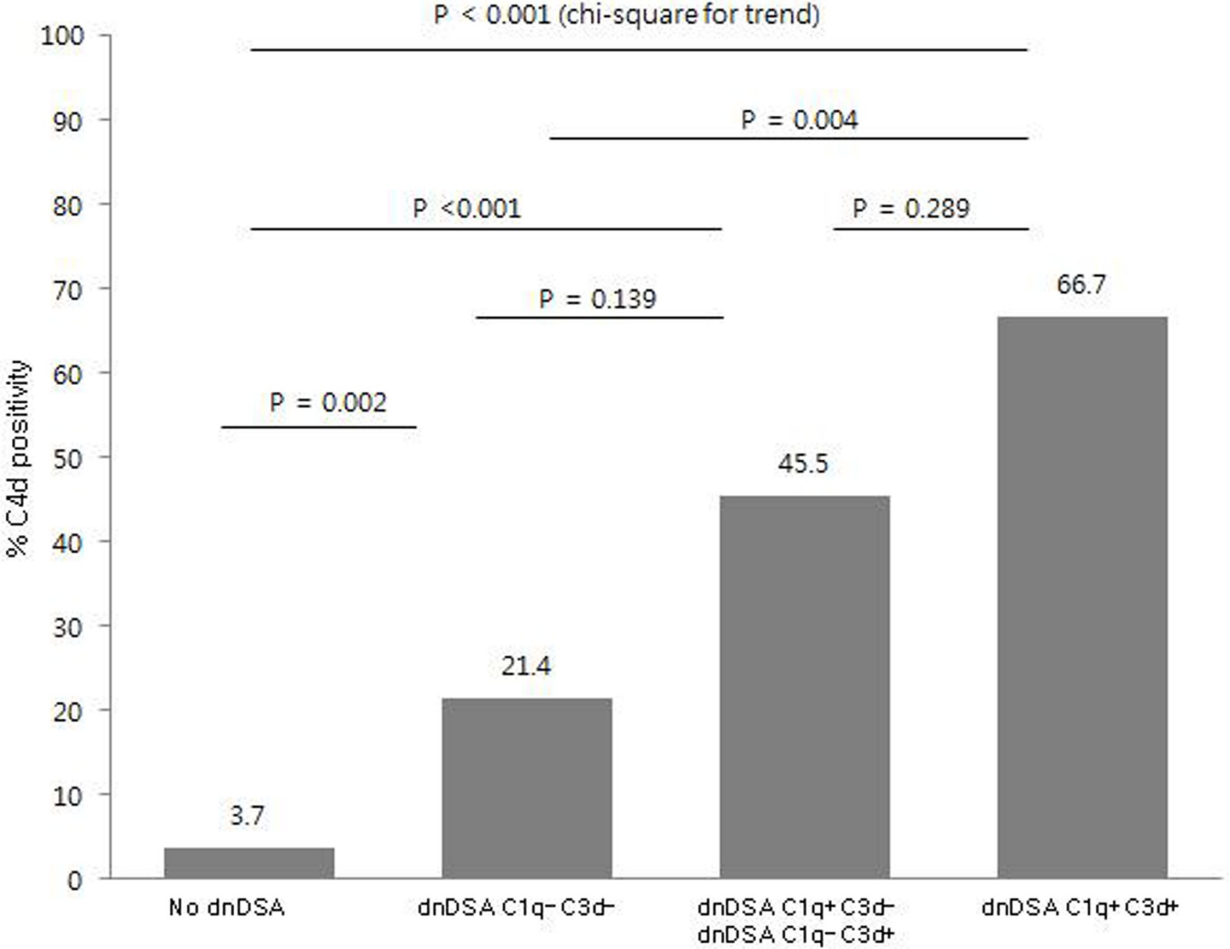
Ratio of C4d deposition according to complement binding activity. Complement binding activity of dnDSA increased the incidence of C4d deposition on biopsy (*P* < 0.001, Chi-square for trend). The comparison of each group was based on Fisher`s exact test. Patients with both C1q+ and C3d+ dnDSA had the highest frequency of C4d deposition (10/15, 66.7%). Abbreviation: dnDSA, de-novo donor specific HLA antibody.

Of 54 dnDSA+ patients, 21 (38.9%) patients had C4d positivity on allograft biopsy. Complement binding activity of dnDSA enhanced C4d positivity: 21.4% of patients with C1q-C3d- dnDSAs, 45.5% of patients with C1q or C3d dnDSAs, and 66.7% of patients with C1q+/C3d+ dnDSAs (*P* < 0.001, Chi-square for trend) (Fig 4). Both C3d+ dnDSA and C1q+ dnDSA were associated with C4d deposition in allograft tissue (*P* < 0.001). There was no significant difference in C4d positivity between patients with C1q+ dnDSAs and those with C3d+ dnDSAs [64.7% (11/17) versus 58.3% (14/24), *P* = 0.684].

Histopathological findings at the time of dnDSA detection were compared according to C1q and C3d positivity (Table 4). In Banff scores, interstitial inflammation (i), total inflammation (ti), chronic glomerulopathy (cg) and C4d score were significantly higher in patients with C1q+ or C3d + dnDSA than those in patients without C1q/C3d dnDSA. However, there was no significant difference between patients with C1q+ dnDSA and those with C3d + dnDSA.

**Table 4:** Banff histology by C1q and C3d status in 161 recipients.

Banff score (mean, SD)	C1q+ dnDSA (N = 17)	C3d+ dnDSA (N = 24)	C1q-C3d- dnDSA (N = 28)	dnDSA- (N = 107)
Glomerulitis (g)	1.76 (1.25) †	1.54 (1.28) †	1.11 (1.13)†	0.36 (0.79)
Tubulitis (t)	1.12 (1.17)	1.13 (1.12)	0.75 (1.08)	0.62 (0.95)
Interstitial inflammation (i)	2.12 (0.78) *†	2.13 (0.85)*†	1.25 (0.93)	1.22 (0.94)
Intimal arteritis (v)	0.18 (0.53)	0.17 (0.48)	0.11 (0.42)	0.10 (0.39)
Arteriolar hyaline thickening (ah)	0.53 (0.87)	0.46 (0.78)	0.18 (0.67)	0.42 (0.91)
Peritubular capillaritis (ptc)	2.12 (1.27) †	1.75 (1.29) †	1.64 (1.37) †	0.25 (0.67)
Total inflammation (ti)	2.24 (0.75)*†	2.29 (0.75)*†	1.57 (0.92)	1.23 (0.95)
Chronic glomerulopathy (cg)	1.24 (1.52)*†	1.13 (1.48)*†	0.36 (0.83)	0.07 (0.45)
Chronic changes in tubules (ct)	1.12 (0.93)	1.21 (0.88)	0.96 (0.74)	0.80 (1.00)
Chronic changes in the interstitium (ci)	1.12 (0.93)	1.21 (0.88)	0.96 (0.74)	0.77 (1.01)
Chronic changes in vessels (cv)	0.12 (0.49)	0.08 (0.41)	0.00 (0.00)	0.01 (0.09)
Mesangial matrix increase (mm)	0.88 (0.93)	0.79 (0.84)	0.50 (0.75)	0.43 (0.72)
C4d score	1.94 (1.48)*†	1.79 (1.47)*†	0.77 (1.27) †	0.10 (0.49)
Historogic diagnosis (N, %)				
MVI	13 (76.5) †	18 (75.0) †	19 (67.9) †	21 (19.6)
IFTA	2 (11.8)	6 (25.0)	9 (32.1)	23 (21.5)
TCMR	6 (35.3)	8 (33.3)	4 (14.3)	27 (25.2)

Abbreviations: MVI, microvascular inflammation (g+ptc ≥ 2); TCMR, T cell-mediated rejection; IFTA, interstitial fibrosis and tubular atrophy.

* *P* < 0.05 compared with C1q-C3d- dnDSA group.

^†^ *P* < 0.05 compared with dnDSA- group.

### Post-biopsy graft survival

**Fig 5:**
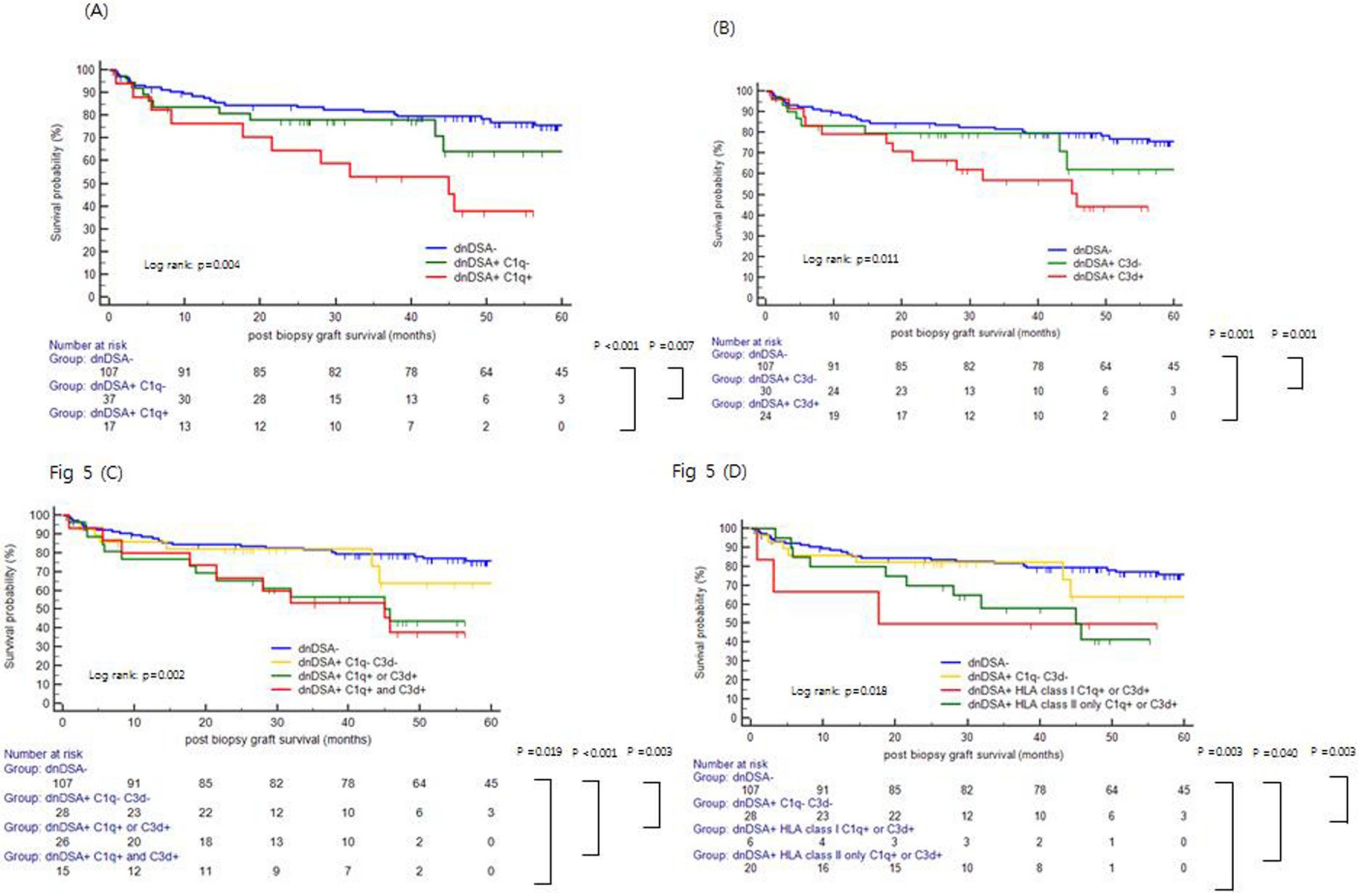
Post-biopsy graft survival according to complement binding activity. **(A)** C1q binding dnDSA correlated with post-biopsy graft survival (Log rank, *P* = 0.005). **(B)** C3d binding dnDSA correlated with post-biopsy graft survival (Log rank, *P* = 0.012). **(C)** The presence of complement binding dnDSA correlated with post-biopsy graft survival (Log rank, *P* = 0.002). In this comparison, fifteen patients with C1q + and C3d + dnDSA are overlapped as they also belonged to the group of dnDSA+ C1q+ or C3d+. **(D)** Patients with complement binding HLA class I ± II dnDSA showed early graft failure.

**Fig 6:**
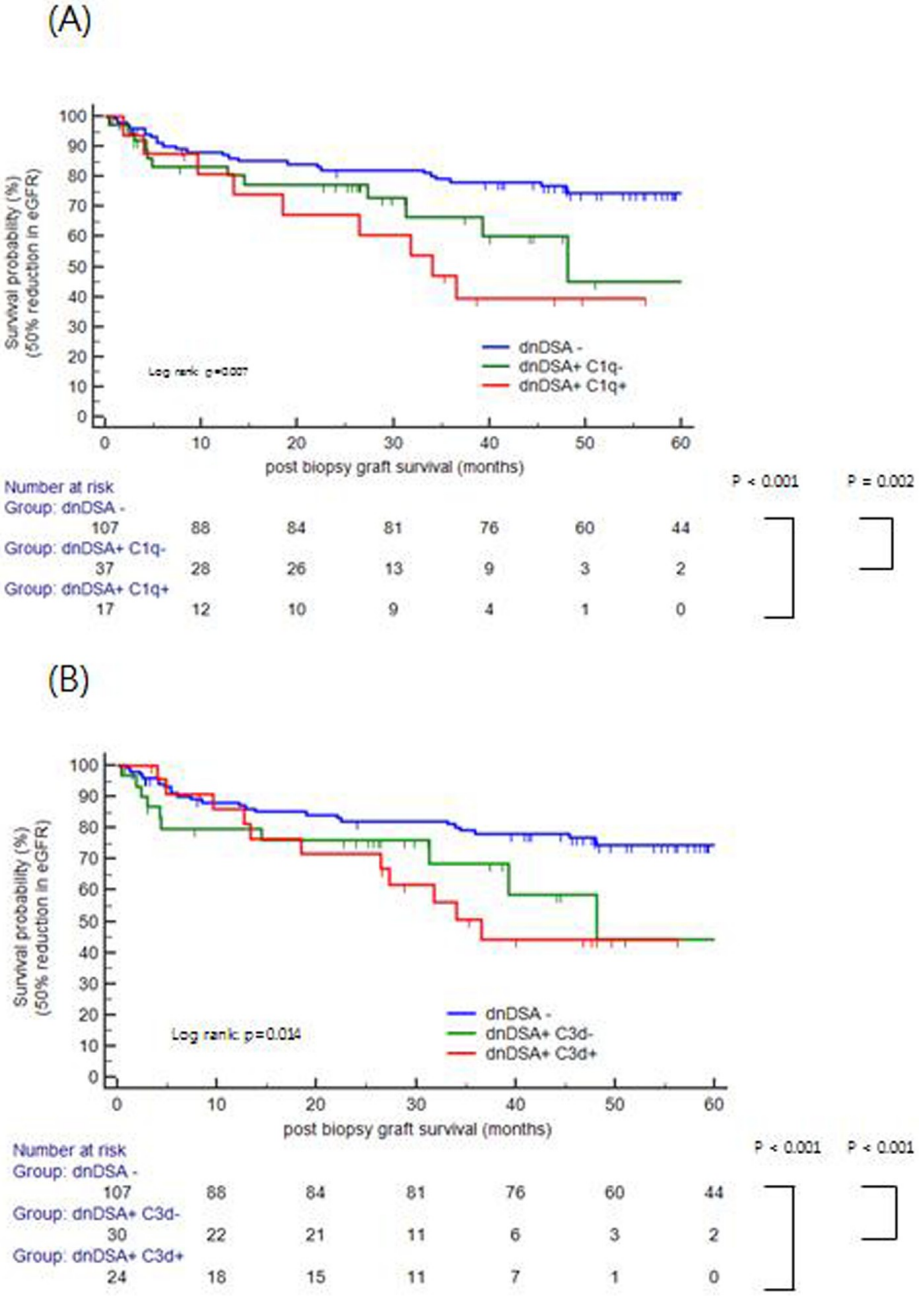
Post-biopsy graft survival defined as a 50% reduction of estimated glomerular filtration rate (eGFR) according to complement binding activity. C1q+dnDSA (A) and C3d+dnDSA (B) showed associated with eGFR decline (Log rank, *P* = 0.007 for C1q and *P* = 0.014 for C3d).

Forty-four patients lost their graft due to various reasons, including rejection (n = 32), interstitial fibrosis and tubular atrophy (n = 6), and recurrence of primary renal disease (n = 4), etc. Table 5 displays risk factors using Cox proportional hazards regression and competing risk models. In a univariate analysis, significant predictors for post-biopsy graft survival were serum creatinine (≥ 2.5 mg/dl) and proteinuria (≥ 2.0 g/g for spot urine protein to creatinine ratio) at the time of biopsy, dnDSA, C1q+, C3d+ and microvascular inflammation (MVI) in biopsy. In multivariate analysis via the Cox proportional hazards model, serum creatinine (≥ 2.5 mg/dL) and proteinuria (≥ 2.0 g/g) at the time of biopsy were associated with graft survival. However, dnDSA or complement binding dnDSA did not reach statistical significance in either the multivariate Cox model or competing risk analyses. Both C1q+ and C3d+ dnDSA showed poor post-biopsy graft survival [Log rank, *P* = 0.004 for C1q, Fig 5A; *P* = 0.011 for C3d, Fig 5B]. The presence of complement binding dnDSA was correlated with poor post-biopsy graft survival [Log rank, *P* = 0.002, Fig 5C]. In detail, complement binding HLA class I ± II dnDSA showed the poorest post biopsy survival in Fig 5D. Although no significance was found (*P* = 0.642), the graft failure occurred earlier in patients with complement binding HLA class I ± II dnDSA compared to patients with complement binding HLA-class II only dnDSA. We also analyzed the impact of C1q and C3d binding dnDSA in post biopsy graft survival stratified for AMR status, but there was no statistical significance (S1 Fig). When we analyzed 50% reduction in estimated glomerular filtration rate (eGFR) as a surrogate marker of long-term allograft survival, complement-binding dnDSA showed also association with eGFR decline (Fig 6).

**Table 5:** Cox regression analysis for graft failure associated risk factors and competing risk analysis.

Cox regression	HR^*^ (95% CI)		P-value	SHR^†^ (95% CI)		P-value
Univariate analysis						
Recipient age, year	1.00	(0.98–1.03)	0.978	1.00	(0.97–1.02)	0.807
Sex (male)	1.67	(0.74–3.74)	0.214	1.62	(0.72–3.64)	0.243
Retransplantation	1.04	(0.25–4.31)	0.955	1.09	(0.29–4.19)	0.895
No. mismatch HLA A/B/DR	1.05	(0.88–1.26)	0.566	1.05	(0.87–1.28)	0.584
Cr (≥ 2.5 mg/dL) ^††^	4.45	(2.46–8.03)	<0.001	4.58	(2.54–8.24)	<0.001
Proteinuria (≥ 2.0 g/g) ^††^	5.61	(3.11–10.14)	<0.001	5.11	(2.83–9.21)	<0.001
Deceased donor	1.03	(0.55–1.91)	0.937	0.98	(0.53–1.81)	0.945
dnDSA	2.21	(1.20–4.05)	0.011	2.19	(1.19–4.03)	0.012
C1q+ dnDSA	2.90	(1.43–5.89)	0.003	3.05	(1.58–5.87)	0.001
C3d+ dnDSA	2.82	(1.46–5.43)	0.002	2.97	(1.58–5.57)	0.001
C1q+ C3d + dnDSA	2.78	(1.33–5.81)	0.006	2.93	(1.51–5.67)	0.001
C4d+	1.64	(0.81–3.33)	0.172	1.73	(0.90–3.34)	0.101
MVI	1.86	(1.03–3.37)	0.040	1.91	(1.05–3.47)	0.033
Multivariate analysis						
Cr (≥ 2.5 mg/dL) ^††^	3.45	(1.85–6.43)	<0.001	3.64	(1.95–6.78)	<0.001
Proteinuria (≥ 2.0 g/g) ^††^	3.82	(2.01–7.29)	<0.001	3.49	(1.84–6.62)	<0.001

*Hazard ratios are from Cox proportional regression model (n = 161 patients and 44 graft failure)

^†^Subdistribution hazard ratios are from competing risk models when death was treated as competing risk (n = 161 patients, 12 deaths and 44 graft failure)

^††^at the time of renal biopsy

Abbreviations: MVI, microvascular inflammation (g+ptc ≥ 2); dnDSA: de novo donor-specific HLA antibodies; Cr, serum creatinine

## Discussion

In present study, we retrospectively detected complement-binding activity of DSA detected at the time of biopsy in 161 pretransplant DSA-negative patients. Lately, it has been shown that DSA monitoring combined with C1q or C3d binding activity provides more predictive value for allograft outcome compared to the non-complement binding DSA [15, 18–22, 32–36]. This study evaluated both C1q and C3d activity of DSA and our results showed that complement-binding assay further stratified dnDSA+ patients at risk of poor graft outcomes, with C1q+/C3d+ HLA class I dnDSA having the highest incidence for AMR.

We found that 33.5% (n = 54) of recipients had dnDSA and 48.1% of dnDSAs (n = 26) had complement-binding activity. This result is similar to a previous study showing that 55.4–67.5% of dnDSAs have complement binding activity [24, 34]. When we stratified patients by HLA class specificity, only 26.3% of HLA class I-reactive dnDSA had complement binding activity while the majority (58.6%) of HLA class II dnDSA had C1q or C3d positivity. This result supports a previous study showing that 70.3% of HLA class II-reactive DSA binds C3d [34]. Higher IgG MFI of HLA class II dnDSA than HLA class I (shown in Fig 2) might lead to greater complement binding dnDSA as discussed above. In this study, we detected dnDSA by using LABscreen assay. Therefore, we cannot rule out that dnDSA might be detected using Immucore assay only. However, a previous study has shown agreement exceeding 90% between laboratories and kits [37]. Therefore, this difference would not have had a significant impact on the results. In comparison of two complement-binding assays, C1q and C3d assays showed moderate strength of agreement (kappa coefficient of 0.58). The disagreement of C1q and C3d assay was already observed in a previous report [24]. The specific difference or functional characteristics of antibodies in the assays needs to be addressed in the future [24].

Correlation between MFI value and C1q/C3d positivity has been previously reported [34, 38, 39]. Similarly, we found that complement binding activity was closely related to strength of HLA antibodies. The relationship seems appropriate as higher MFIs correlate with higher antibody titers, making greater antibody binding and enhancing multivalent C1q binding avidity [40]. However, using MFI as a surrogate for determining complement activating capacity of HLA antibodies remains debatable as not all studies have shown this relationship [16, 41, 42]. In our results, complement binding dnDSAs had higher MFI values than C1q- or C3d- dnDSAs, especially HLA class II dnDSA. However, C3d+ dnDSA against HLA class II showed a large overlap between MFI values while a clear threshold could not be determined, similar to a previous report showing poor correlation between IgG MFI and C3d results [24].

In present study, both C1q+ and C3d+ dnDSAs were associated with more C4d staining. Complement binding activity of dnDSA enhanced the incidence of AMR: 25.0% in C1q-C3d-, 36.4% in C1q+/C3d- or C1q-/C3d+, and 60.0% in C1q+/C3d+ patients (*P* < 0.001). However, in comparison between C1q and C3d assays, C3d assay could not significantly differentiate AMR in dnDSA+ patients. Although the incidence of AMR was not significantly different between C1q+ and C3d+ patients [64.7% (11/17) versus 45.8 (11/24), *P* = 0.238], these findings were not concordant with those of previous studies which showed lower proportion of C3d positivity and better prognostic predictability with C3d assay [22, 24]. This might be due to different study population including higher proportion of dnDSAs against HLA class II antigen or different post-KT duration until allograft biopsy. In our study, nine patients with C1q-C3d+ dnDSA had only HLA class II dnDSA and two of them were diagnosed as AMR. The discrepancy in results could be due to interference in C1q assay, such as the presence of denatured HLA antigen, complement interference or the detection of potentially non-significant antibody [24, 43]. Further studies are needed to confirm the specific difference or functional characteristics.

However, C1q+ dnDSAs was better diagnostic predictors for AMR than total dnDSA in present study. Especially, C1q+ HLA class I ± II dnDSA was the best predictor for AMR (odds ratio: 27.2). However, clinical outcomes were not significantly different between C1q+ and C3d+ dnDSA-positive patients.

Monitoring of post-transplant serum DSA is noninvasive and serial test is easy compared to C4d staining. C4d is a specific marker for AMR. However, it lacks sensitivity, particularly for late AMR [44]. DSA detection has identified patients with high risk for graft failure despite C4d negativity in biopsy [45, 46]. In line with these findings, although we cannot confirm the independent predictive value of C1q and C3d, our results support the notion that post-transplant DSA monitoring with complement binding assay would be a better prognostic marker after kidney transplantation than C4d stain.

This study has some limitations. Although our results demonstrated significant association between complement binding dnDSA and AMR, the results were derived from a selected population undergoing indication graft biopsy with different follow-up durations and might not be extended to the general population of kidney recipients. In addition, we could not specify dnDSAs against DQ alpha and beta protein though AMR due to DQ alpha protein-specific HLA antibody was reported [47].

In conclusion, our results suggest that detection of complement binding activity using both C1q and C3d assays can be a further prognostic marker for predicting AMR and allograft outcome in dnDSA+ kidney transplant patients. Thus, C1q or C3d binding assay might be used as a complementary test to conventional IgG assay in assessing the risk of AMR.

## Supporting information

S1 Fig Post-biopsy graft survival stratified for AMR status. Post biopsy graft survival according to the C1q positivity in patients with AMR (A) and without AMR (B). Post biopsy graft survival according to the C3d positivity in patients with AMR (C) and without AMR (D). Although there was no statistical significance, patients with C1q+ dnDSA and C3d+dnDSA showed inferior post biopsy graft survival, especially in AMR negative group (Log rank, *P* = 0.116 for C1q and *P* = 0.234 for C3d).
(TIF)
