# Supplementary figures and images for: Clinical impact of complement (C1q, C3d) binding De Novo donor-specific HLA antibody in kidney transplant recipients
Source: PLoS One. 2018 Nov 14;13(11):e0207434. doi: 10.1371/journal.pone.0207434 (PMC6235372; doi:10.1371/journal.pone.0207434)

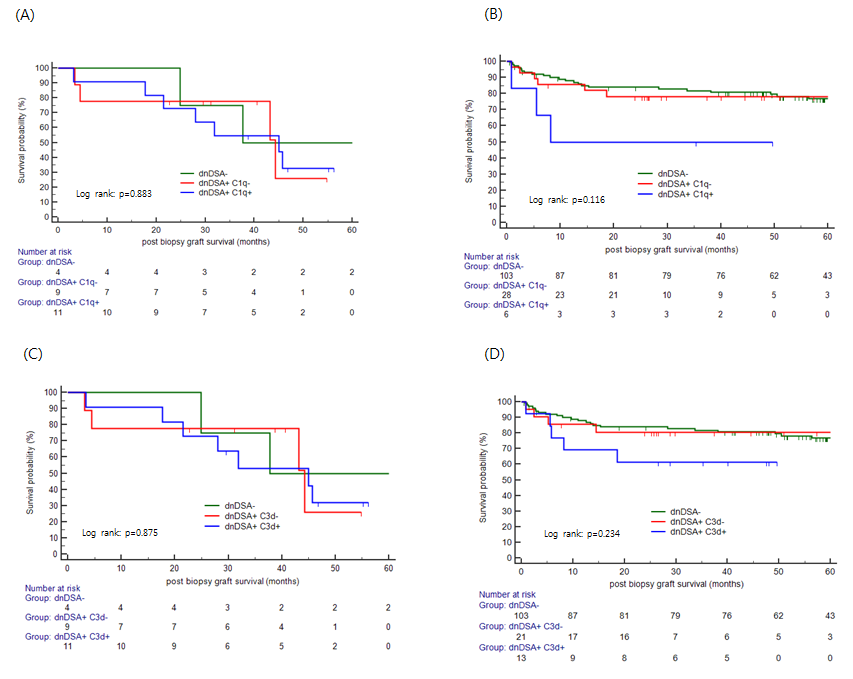

Supplement: S1 Fig — Post biopsy graft survival according to the C1q positivity in patients with AMR (A) and without AMR (B). Post biopsy graft survival according to the C3d positivity in patients with AMR (C) and without AMR (D). Although there was no statistical significance, patients with C1q+ dnDSA and C3d+dnDSA showed inferior post biopsy graft survival, especially in AMR negative group (Log rank, P = 0.116 for C1q and P = 0.234 for C3d). (TIF) [file pone.0207434.s001.TIF]
